# Temporal Geospatial Analysis of COVID-19 Pre-Infection Determinants of Risk in South Carolina

**DOI:** 10.3390/ijerph18189673

**Published:** 2021-09-14

**Authors:** Tianchu Lyu, Nicole Hair, Nicholas Yell, Zhenlong Li, Shan Qiao, Chen Liang, Xiaoming Li

**Affiliations:** 1 Department of Health Services Policy and Management, Arnold School of Public Health, University of South Carolina, Columbia, SC 29208, USA; tlyu@email.sc.edu (T.L.); hairnl@mailbox.sc.edu (N.H.); 2 Department of Statistics, College of Arts and Sciences, University of South Carolina, Columbia, SC 29208, USA; yelln@email.sc.edu; 3 Department of Geography, College of Arts and Sciences, University of South Carolina, Columbia, SC 29208, USA; zhenlong@mailbox.sc.edu; 4 Department of Health Promotion, Education, and Behavior, Arnold School of Public Health, University of South Carolina, Columbia, SC 29208, USA; shanqiao@mailbox.sc.edu (S.Q.); xiaoming@mailbox.sc.edu (X.L.)

**Keywords:** COVID-19, healthcare disparities, social determinants of health, spatial analysis, Post-Acute Sequelae of SARS-CoV-2 infection

## Abstract

Disparities and their geospatial patterns exist in morbidity and mortality of COVID-19 patients. When it comes to the infection rate, there is a dearth of research with respect to the disparity structure, its geospatial characteristics, and the pre-infection determinants of risk (PIDRs). This work aimed to assess the temporal–geospatial associations between PIDRs and COVID-19 infection at the county level in South Carolina. We used the spatial error model (SEM), spatial lag model (SLM), and conditional autoregressive model (CAR) as global models and the geographically weighted regression model (GWR) as a local model. The data were retrieved from multiple sources including USAFacts, U.S. Census Bureau, and the Population Estimates Program. The percentage of males and the unemployed population were positively associated with geodistributions of COVID-19 infection (*p* values < 0.05) in global models throughout the time. The percentage of the white population and the obesity rate showed divergent spatial correlations at different times of the pandemic. GWR models fit better than global models, suggesting nonstationary correlations between a region and its neighbors. Characterized by temporal–geospatial patterns, disparities in COVID-19 infection rate and their PIDRs are different from the mortality and morbidity of COVID-19 patients. Our findings suggest the importance of prioritizing different populations and developing tailored interventions at different times of the pandemic.

## 1. Introduction

Coronavirus disease 2019 (COVID-19), caused by the Severe Acute Respiratory Syndrome Coronavirus 2 (SARS-CoV-2), is a highly contagious disease that has caused widespread panic and concern across the globe. COVID-19 was the third leading cause of death in 2020. The death rate increased by 15.9% from 2019 to 2020 [1]. As of September 2020, there have been 41 million confirmed cases and 660 thousand deaths due to COVID-19 in the USA [1,2,3]. Additionally, COVID-19 has had a profound impact on social life and the economy, as closing businesses and social distancing have been common practices to slow the spread of the disease. The U.S. real GDP decreased by 3.5% in 2020 and was projected to lose at least $3.2 trillion due to COVID-19 in a two-year course [4,5].

The burdens of COVID-19 have not been borne equally. Some populations face increased risk for COVID-19 morbidity and mortality [6]. Many studies have reported disparities in the clinical outcomes of patients with COVID-19. For example, studies using inpatient data found severe disease progression and poor clinical outcomes of COVID-19 patients to be associated with a set of underlying medical conditions (e.g., hypertension, diabetes, asthma, and heart, liver, and respiratory illnesses), demographics (e.g., male, older age, race/ethnic minority), and social determinants of health (SDOHs) (e.g., lower education and income) [7,8,9,10,11]. A study based on a large cohort in Louisiana comprised of 3,481 COVID-19 patients reported that 76.9% of the hospitalized cases and 70.6% of the death cases were among black patients, whereas only 31% of the state’s population is black [12]. While these studies have provided a critical evidence base of disparities in COVID-19 clinical outcomes and implications for medical care for addressing the disparities, they offered limited implications for disparities in the risk of exposure to COVID-19 for the following reasons. First, the findings of these studies are applicable for hospitalized patients but may not be generalizable for outpatients, individuals with mild symptoms, and asymptomatic individuals since these studies are based on inpatient data. The omission of outpatients and individuals with laboratory-confirmed COVID-19 infections but no clinic visits will harm the potential opportunity of exploring risk factors for these populations [13]. Second, using disease severity as the outcome variable does not provide information on SARS-CoV-2 infection and transmission. For example, SARS-CoV-2 transmits more easily in regions with a large proportion of younger people, yet the elderly were found to be at a higher risk of developing poor clinical outcomes [14].

Therefore, it is equally important to curate an evidence base for disparities in the risk of exposure to COVID-19 and the pre-infection determinants of risk (PIDRs) (e.g., demographics, socioeconomics, and prevalence of diseases related to COVID-19 infection) [15,16,17,18,19,20]. Such an evidence base can be used for understanding disease transmission patterns, identifying vulnerable populations, and proactively mitigating disparities in future pandemics [21]. Existing studies have reported demographic and socioeconomic factors to be related to disparities in the risk of exposure to COVID-19. Different combinations of those determinants lead to different health attributes (e.g., health behaviors and physical conditions), thus influencing the spread of the virus. For example, high-deprivation areas have higher rates of hospitalization and testing [17]. People with a higher income are more likely to engage in self-protecting behavior during the COVID-19 pandemic [18]. Another study reported that the behaviors of wearing masks and using hand hygiene are associated with the female sex and a higher education level among students in the Chinese population [19]. In a primary care cohort, researchers observed a higher risk of COVID-19 infection among people aged 40–64 years, of the male sex, of the black race, and living in urban areas [15]. Incorporating census tract level data with the COVID-19 dataset, Hawkins and colleagues examined the association between socioeconomic indicators and COVID-19 cases at the county level across the USA and found a lower education level and a higher percentage of black residents to be risk factors for the infection [16].

To further explore the associations between PIDRs and COVID-19 transmission, geospatial information is needed. Geographic differences exist across states, counties, and communities in the timing of the SARS-CoV-2 introduction, which are further characterized by population density, local policies, and population composition [14]. Particularly, understanding PIDRs and their geospatial epidemiology is urgently needed for rural states, such as South Carolina, that have a disproportionally low healthcare capacity and high disease burden. It may also provide timely information for post-COVID-19 care, given the emerging reports on the heterogeneity of symptoms in individuals with Post-Acute Sequelae of SARS-CoV-2 infection (PASC) [22,23]. Although the spatially dynamic nature of infectious diseases (e.g., different spatial patterns of transmission) makes geospatial analysis a valuable tool to unveil the epidemiology [24,25,26,27], there have been limited studies reporting the geospatial characteristics of PIDRs [14,28,29,30,31]. Several studies have reported minority status, age, and other social vulnerabilities to be associated with a higher COVID-19 infection, yet spatial patterns were generally not included in the statistical models as independent variables [28,30,31]. Fortaleza and colleagues used multivariate regression and found that population density and distance from the state capital are robust predictors of COVID-19 prevalence in Brazil [29]. However, the results should be interpreted carefully since the association between population density and COVID-19 infection could be influenced by factors such as different policies being applied to smaller regions [32]. Another study built a correlation matrix between socioeconomic determinants and COVID-19 case rates across the USA and found population density to be highly correlated with COVID-19 prevalence [14].

Although the above studies have collectively suggested possible geospatial characteristics among the disparities in virus transmission, spatial autocorrelation is generally excluded from their statistical models, which limits the statistical power of the findings. The spatial autocorrelation, including global modeling and local modeling approaches, enables the correlation measure of a variable (e.g., PIDRs) with itself across different regions. Spatial global models assume a stationary correlation between a region and its neighbors, whereas spatial local models assume nonstationary correlations between a region and different neighbors. Among a few preliminary studies that adopted spatial autocorrelation, Mollalo and colleagues examined the association between the COVID-19 incidence rate and four county-level explanatory determinants including income inequality, median household income, the percentage of nurse practitioners, and the proportion of the black female population to the total female population across the USA [33]. The authors started with a set of 35 socioeconomic, behavioral, topographic, and demographic explanatory variables. After a stepwise forward procedure and correlation analysis, they choose to keep four of these variables in their final model and found that geographically weighted regression (GWR) models best explained the variations, suggesting the existence of spatial autocorrelation and different vulnerabilities across the counties. Despite the application of highly appropriate geospatial methods, the study could have better interpreted the disparity structure if demographic determinants such as age, sex, and race were included in the analysis. Additionally, because these studies were based on analyses of cross-sectional data, they did not specify whether and how observed relationships between COVID-19 outcomes and PIDRs vary at different points in time as the pandemic evolved. Moreover, there is increased endogeneity in these analyses because they focused on large geographic regions within which different regional policies might have a greater impact on the COVID-19 prevalence as compared with the explanatory variables. Existing evidence suggests that government responses and socioeconomic determinants have played an important role in the transmission of SARS-CoV-2, which differs geographically [34]. Another similar study included demographics but still suffered from the same endogeneity problem [35].

Building on these existing studies, we sought to assess the association between PIDRs (including demographics, socioeconomics, and prevalence of diseases related to COVID-19 infection) and COVID-19 infection at the county level in South Carolina at different timepoints amid the pandemic. The heterogeneity in the virus spread in South Carolina suggests that different PIDRs in certain areas could enhance or inhibit the transmission of COVID-19. Within the smaller geographic scale of one state, the heterogeneous impacts of different regional policies could be largely mitigated, and the multi-source South Carolina surveillance data were sufficient for conducting geospatial analyses. Although there has been no statewide mask mandate in South Carolina, regional mask ordinances covered most of the regions by July 2021 [36]. The findings of this study form an evidence base for temporal geospatial disparities in the risk of exposure to COVID-19 and the associated PIDRs. The identified PIDRs may also shed light on the populations and regions vulnerable to PASC in South Carolina during post-COVID-19 care.

## 2. Materials and Methods

### 2.1. Model Selection

We selected six time windows to represent COVID-19 cases at different times of the pandemic. South Carolina began tracking COVID-19 cases in early March of 2020 and the number of daily new cases began to rise until July 2020 when the daily number of new cases began to fluctuate. We calculated the average cumulated case numbers in a sliding window of seven days (15th ± 3 days) for every month between July and December of 2020.

The US Centers for Disease Control and Prevention (CDC) have provided a list of risk factors of COVID-19 severity such as age and existing medical conditions [37]. As discussed in the introduction, the PIDRs for COVID-19 severity can be very different from the PIDRs for COVID-19 infection. Based on previous studies, Snyder and Parks presented a well-developed risk factor index framework for COVID-19 community vulnerability which was defined as “the potential decrease in the wellbeing of a community before and during/after the pandemic, taking into account health, social, and economic conditions” [38,39,40]. The index is divided into four major sections (e.g., ecological, social, health, and economic) [39]. Inspired by their study and based on data availability in South Carolina, we began with 15 different variables related to the four sections of the index including sex, age, race, median household income, population density, uninsured rate, poverty percentage, high school degree rate, college degree rate, unemployment rate, physical inactivity rate, obesity rate, smoking prevalence, medical doctors per 10,000 people, and nurse practitioners per 10,000 people (Table 1). Among the candidate variables, age, sex, and population density represent ecological variables; uninsured rate, education levels, race, medical doctor abundance, and nurse practitioner abundance are social variables; obesity rate, physical inactivity rate, and smoking prevalence are health variables; and income, poverty rate, and unemployment rate are economic variables.

We then tested multicollinearity across the candidate variables and finetuned the final model with variables including age, sex, race, and socioeconomic variables, unemployment rate, uninsured rate, college degree rate, obesity rate, and nurse practitioner per 10,000 people. Specifically, we excluded the variables (e.g., median household income, population density, poverty percentage, high school degree rate, physical inactivity rate, smoking prevalence, medical doctors per 10,000 people) that were highly correlated with other variables (correlation coefficient >0.7) in this step. We employed this relaxed criterion for two reasons: (1) Because we used spatial regression afterward, multicollinearity would be different after we incorporated spatial autocorrelation. (2) We intended to include as many variables as possible to better represent Snyder and Parks’ index, so that the results could be intuitive and interpretable.

### 2.2. Data Sources

The data sources used in this work varied. The age variable was extracted from U.S. Census Population and Housing Unit Estimates, 2010–2018. Sex, race, and college degree rate were extracted from the U.S. Census Bureau, Population Estimates Program (PEP), and American Community Survey (ACS), updated 1 July 2019. The unemployment rate and poverty rate were retrieved from the U.S. Census Bureau, Small Area Income and Poverty Estimates (SAIPE) Program (2019). The uninsured rate was retrieved from the U.S. Census Bureau, Small Area Health Insurance Estimates (SAHIE) Program (2018). The obesity rate was retrieved from U.S. CDC Diabetes County Data Indicators, 2006–2017. The nurse practitioner number was retrieved from Health Resources & Services Administration (HRSA) Area Health Resources Files, 2017 and 2018. The confirmed cases number of COVID-19 from July 2020 to December 2020 was obtained from USAFacts, which is also the data source that the U.S. CDC uses. Specifically, the case data of a certain date reflect the cumulative totals of that date [41]. Log transformation was applied in the dependent variable and the explanatory variables to normalize skewed data.

### 2.3. Spatial Regression Models

**Figure 1:**
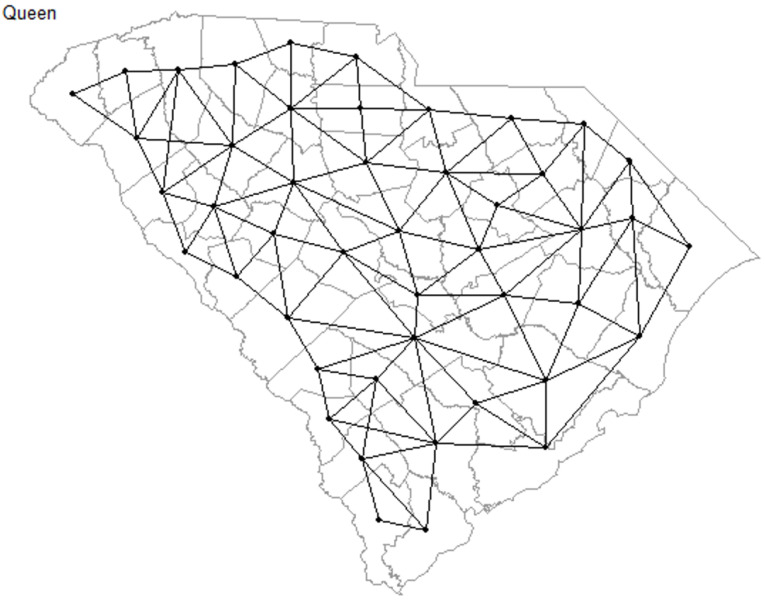
Contiguity Queen neighbors of counties in South Carolina.

We calculated spatial weights using queen contiguity which defines neighbors by the presence of shared edges and vertices. Figure 1 shows the county map of South Carolina with the links between each neighbor (i.e., county). Spatial modeling was used to describe the relationship between the COVID-19 cases and factors at the county level. The following spatial models were used to fit our data: spatial error model (SEM), spatial lag model (SLM), conditional autoregressive (CAR) model, and GWR model. We used SEM to observe spatial autocorrelation between the residuals of neighboring counties, which incorporates spatial effects through the error term. SLM applies spatial dependence by adding a spatially lagged response variable as an additional predictor on the linear model equation. This model assumes that the COVID-19 incidence rate in one county is directly influenced by the COVID-19 incidence rates in its neighboring counties. If positive spatial lag is observed in SLM, it would suggest that COVID-19 incidence rates in neighboring counties covary. The CAR model relies on the conditional distribution of the spatial error terms and assumes the region is a function of its neighbors but not the neighbors of neighbors (i.e., first-order dependency). We used the GWR method to examine the local models, which is based on kernel-weighted regression and allows for parameters to vary spatially [42].

## 3. Results

### 3.1. Distribution of COVID-19 Cases and Covariates

**Figure 2:**
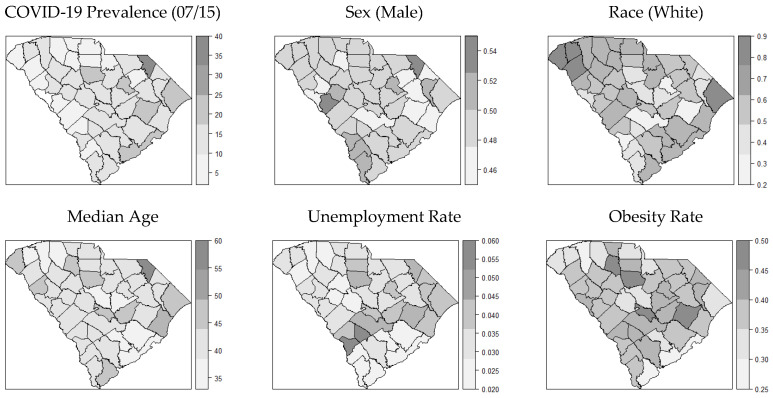

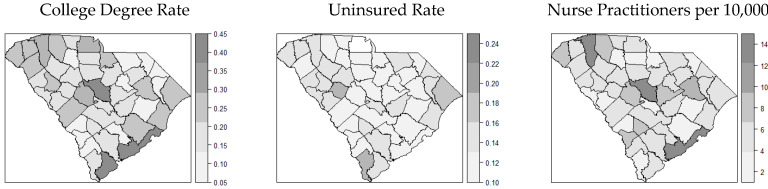
Distribution maps of the variables (in percentages) in the model.

After model selection, the final model contained eight explanatory variables, namely sex, race, age, college degree rate, obesity rate, unemployment rate, uninsured rate, and nursing practitioner abundance. We summarized and showed maps of distributions for all the variables in the model (Figure 2). To make the descriptive map comparison between variables easier, we held back the temporal dimension and used average COVID-19 incidences per 1000 people around July 15th. In Figure 2, we observed some similarities between the distribution of COVID-19 cases and certain demographic and socioeconomic variables. For example, the maps of sex and age were congruent with the map of COVID-19 incidence rate. The map of the obesity rate showed a nearly opposite pattern compared to the map of COVID-19 cases.

### 3.2. Global Models for Spatial Correlation

When considering the temporal dimension, four geospatial models were built to examine the spatial correlation of COVID-19 incidence across the counties in South Carolina including the SEM, SLM, CAR model, and GWR model. The significant results from Moran’s I test (*p* values < 0.05) suggested the existence of spatial autocorrelation. We summarized the coefficients of the variables and corresponding p values for global models in Table 2, Table 3 and Table 4 (i.e., SEM, SLM, and CAR model). All the models were significant at 0.05 level, indicating that spatial autocorrelations did show within the error terms. The percentage of residents who were male and the unemployment rate were statistically significant (*p* values < 0.05) with positive coefficients in the three global models throughout the six time windows, while other variables were not (Table 2, Table 3 and Table 4). Interestingly, the spatial correlations between COVID-19 cases and the percent of residents who were white or obese, respectively, flipped over the course of the pandemic. Earlier in the pandemic, white race was not statistically correlated with COVID-19 cases. Later in the pandemic, beginning in December, it was positively correlated with COVID-19 cases. The obesity rate was negatively correlated with COVID-19 cases as early as July but became positively correlated during the months October through December in SEM and CAR, yet this pattern did not show in SLM.

### 3.3. Local Models for Spatial Correlation

**Figure 3:**
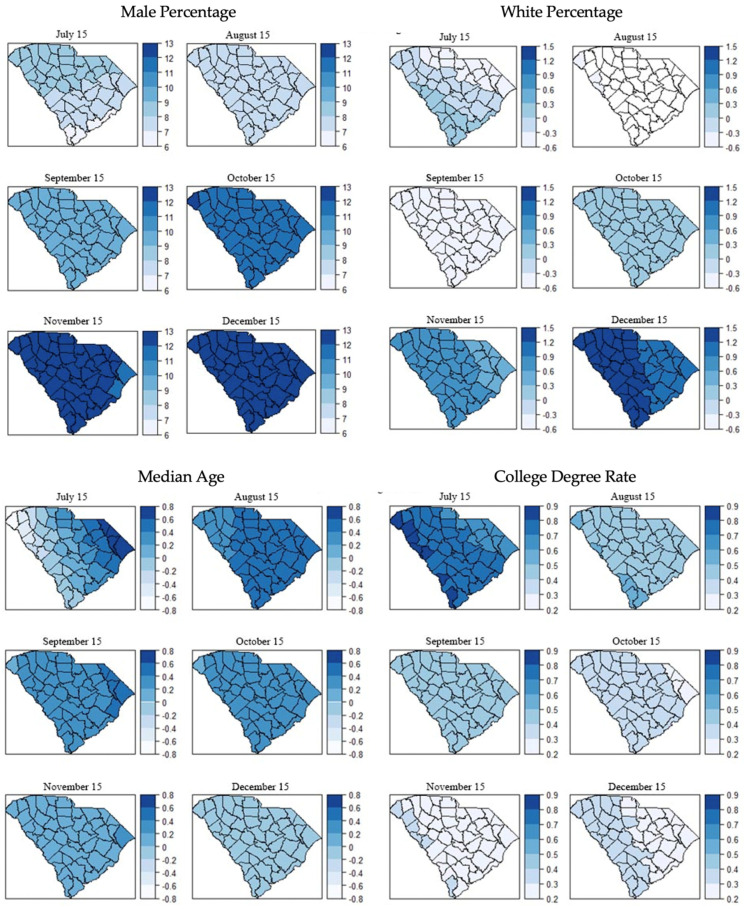

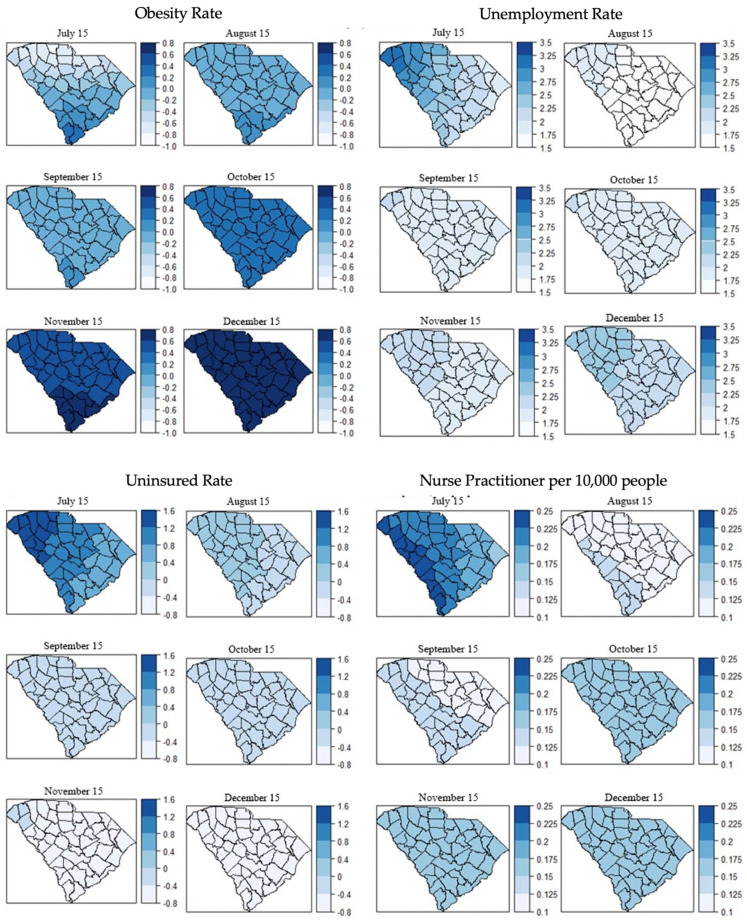
Monthly geographic distribution of local coefficient estimates of GWR models for COVID-19 incidence rate associated with the explanatory variables between 15 July 2020 and 15 December 2020.

The results of the GWR model are summarized in Table 5. In the GWR models, the calculated bandwidths were 60.87 km for July 15 and 154.41 km for the rest of the time points. Taking July 15 as an example, the GWR model possessed the lowest Akaike Information Criterion (AIC) value of 51.24, compared to the global models such as SEM (AIC = 61.59), SLM (AIC = 58.72), and the CAR model (AIC = 59.39) (Table 6). A smaller AIC indicates a better fit when compared with other models that were built on the same data. These two findings collectively suggest highly localized spatial correlations at the beginning of the pandemic, yet this effect started to decline as the pandemic evolved. Figure 3 shows the geographic distribution of local coefficient estimates of GWR models for COVID-19 incidence rate associated with each explanatory variable. For each explanatory variable, we can observe a clear trend suggesting that the heterogeneity among coefficients became homogeneity throughout the time.

## 4. Discussion

In this geospatial study, we adopted the socioecological vulnerability index from Snyder and Parks and compiled 15 variables within four categories of the index which could potentially explain the geographic patterns of COVID-19 transmission in SC [39]. Our study resulted in three principal findings. First, our study demonstrated the spatial autocorrelations of COVID-19 incidence at the county level in SC. The results from global models and local models were consistent with the initial observation of the distribution maps of covariates. Second, some PIDRs (e.g., male percentage, unemployment rate) had consistent spatial correlations with COVID-19 incidence over time while some other PIDRs (e.g., percentage of the white population, obesity rate) showed divergent spatial correlations at different times of the pandemic, suggesting a critical role of the temporal dimension in the geospatial epidemiology of COVID-19 transmission. Third, the geospatial effect of PIDRs was strong at the beginning of the pandemic and started to decline as the infection cases continued to surge, suggesting the importance of early identification of critical PIDRs and timely intervention for possible future outbreaks of infectious diseases.

Aligned with existing studies [28,30], two PIDRs (e.g., male percentage and unemployment rate) were found to be significantly associated with a higher risk of COVID-19 infection in global models (e.g., SEM, SLM, and CAR). The higher risk of COVID-19 infection among the male population can be explained by several sex-related factors [43]. Genetically, males have a higher expression of angiotensin-converting enzyme-2 (ACE2), which could be the receptor for SARS-CoV-2 [44,45]. The immunological response of SAR-CoV-2 may be different between males and females [46,47]. In addition, females have been found to have a more responsible attitude of health behaviors towards COVID-19 than males [19,48]. A higher unemployment rate reflects a higher socioeconomic vulnerability of COVID-19 infection. People with the ability to work from home are less likely to be infected because of higher job security [49,50]. Interestingly, our results are different from an existing study from Johnson et al. [51]. They found unemployment to be a protective feature of COVID-19 infection and argued that it might be related to the lack of transportation among the unemployed. The role of unemployment in COVID-19 transmission needs further investigation.

We found that the white population was not statistically correlated with COVID-19 incidence from July to October and became positively correlated with COVID-19 incidence (all *p* < 0.01 for SEM, SLM, CAR) in December. To the best of our knowledge, this finding has not been previously reported. We suspect that this finding is related to the fact that the COVID-19 incidence rate was higher in large metropolitan areas (e.g., urban, suburban) early on in the pandemic (i.e., March–May 2020) and diffused to small and nonmetropolitan areas, where proportions of white people are higher, later [31]. Among the 26 counties that are classified as metropolitan areas in South Carolina, only three have a white population of less than 50%, and five have a white population of less than 60% [52,53]. Previous studies found that racial minorities had a higher risk of COVID-19 infection [28,30,33], but these findings have not been tested or interpreted by the temporal dimension of the pandemic. Cunningham and Wigfall reported that racial attitudes towards COVID-19 had a significant impact on the likelihood of infection and mitigated the effect of racial difference, which also could explain our finding [54]. In addition, our result could be related to the finding that a higher proportion of white people took COVID-19 tests than other races in the latter months [55]. Median age, college degree rate, obesity rate, uninsured rate, and NP abundance were not statistically correlated with the COVID-19 infection rate.

Our findings suggest that early measures could be related to the transmission of COVID-19 since the geographic differences in COVID-19 infection reduced over time, indicated by the decreasing AIC values across models longitudinally (Table 6). The decrease in AICs of local model (i.e., GWR model) over time indicated the persistence of the nonstationary spatial autocorrelation. Although the GWR models have lower AIC values compared with the global models, the coefficients of the variables in GWR models did not vary substantially, indicating small nonstationary effects. The small ranges of the coefficients geographically could be related to the insufficient granularity of the county-level data considering the study sample of South Carolina. Nevertheless, it is very interesting that the regional variances were decreasing over time within the study time frame.

This study is among the first to examine geospatial patterns in COVID-19 infection as well as PIDRs. Most studies have focused on patients with different levels of severity with COVID-19, which limits opportunities for examining possible disparities and PIDRs in COVID-19 infection [56]. For example, older adults, people with certain medical conditions, and pregnant women were found to be associated with a higher risk of severe illnesses of COVID-19, while our study found that the male population and unemployment rate were risk factors of COVID-19 infection [56]. Intuitively, the PIDR set for severe illnesses of COVID-19 is related to the physical condition of patients and the PIDR set for COVID-19 infection is jointly influenced by demographic and socioeconomic factors. Compared with PIDRs for severe illnesses, PIDRs for infection are highly sensitive to geographic regions and temporal dynamics of the pandemic because the transmission of COVID-19 is related to the activity of people. PIDRs for COVID-19 infection provide important information for developing interventions on targeted populations who share the same PIDRs at the beginning of the pandemic, which is imperative for containing the early-stage transmission and potential consequences in future infectious disease outbreaks.

Our study has several limitations. First, we did not use longitudinal measures of PIDRs due to limited surveillance data. Second, we used reported cases as a measure of COVID-19 prevalence. This measure could be potentially biased because testing rate and test positivity were not considered due to unavailable surveillance data. For example, data for COVID-19 testing rates for each race were not available for examining the racial differences [57,58,59]. Third, due to the limited data access, we used county-level data in this study whereas using zip code-level data would have offered a better granularity of data in the statistical models. Fourth, mobility patterns have been identified as an important factor for COVID-19 transmission, which is not accounted for due to the limited data availability [60]. Fifth, our methodology does not include the contrast between restrictions and temporary spatial patterns. Thus, implications resulted from temporal patterns should be discussed with caution. At last, variables used in this study may not be exhaustive in terms of all possible contributing factors of COVID-19 infection as this work is based on the framework from Snyder and Parks. Future studies could integrate variables such as the Social Vulnerability Index (SVI) for exploring the negative effects in communities towards hazardous events [61,62,63].

## 5. Conclusions

Our study found that the geospatial distribution of COVID-19 incidence was constantly influenced by several key PIDRs including male percentage and unemployment. PIRDs such as white percentage and obesity rate were negatively correlated with COVID-19 incidence at the beginning of the pandemic and then became positively correlated with COVID-19 incidence. These identified PIDRs are different from those found to be associated with poor clinical outcomes (e.g., severity and mortality) of patients who are engaged with medical care. Our study found disparities in COVID-19 transmission and suggested newly identified temporal dynamics in specific PIDRs such as white percentage and obesity rate. These findings are subject to biases caused by limited data access and should be considered provisional guidelines to the temporal geospatial epidemiology of COVID-19 transmission and underlying PIDRs of the pandemic in South Carolina.

## Figures and Tables

**Table 1:** Candidate explanatory variables and definitions.

Theme	Variable	Definition
Ecological	Age	Median age
	Sex	Percentage of male population to the total population
	Population density	Population per square mile
Social	Uninsured rate	Percentage of population under 65 years old without health insurance
	High school degree rate	Percentage of population with a high school or higher degree
	College degree rate	Percentage of population with a college or higher degree
	Race	Percentage of white population to the total population
	Medical doctor abundance	The number of medical doctors per 10,000 people
	Nurse practitioner abundance	The number of nurse practitioners per 10,000 people
Health	Obesity rate	Percentage of obese population (i.e., individuals whose BMI is 30 or higher)
	Physical inactivity rate	Percentage of population not engaging in physical activity regularly
	Smoking prevalence	Percentage of population who smoke cigarettes regularly (i.e., have smoked at least 100 cigarettes in their life and currently smoke at least one cigarette a day)
Economic	Income	Median household income
	Poverty rate	Percentage of population in poverty
	Unemployment rate	Percentage of unemployment population

**Table 2:** Coefficients of the explanatory variables in SEM.

	July 15		August 15		September 15	
	Coef.	*p* Value	Coef.	*p* Value	Coef.	*p* Value
Male percentage	9.26	* 0.014	7.72	* 0.013	9.65	** 0.002
White percentage	0.21	0.970	−0.06	0.910	−0.05	0.966
Median age	−0.08	0.910	0.16	0.793	0.03	0.960
College degree rate	0.53	0.273	0.39	0.313	0.45	0.228
Obesity rate	−2.17	0.198	−0.76	0.589	−0.25	0.846
Unemployment rate	2.88	** 0.003	2.07	** 0.009	2.19	** 0.004
Uninsured rate	1.91	0.052	0.49	0.539	0.12	0.875
NP abundance	0.08	0.575	0.04	0.737	0.07	0.490
	**October 15**		**November 15**		**December 15**	
	**Coef.**	***p* Value**	**Coef.**	***p* Value**	**Coef.**	***p* Value**
Male percentage	11.30	** 0.000	11.92	** 0.000	11.84	** 0.000
White percentage	0.35	0.407	0.73	0.069	1.17	** 0.003
Median age	0.09	0.855	−0.03	0.952	−0.19	0.694
College degree rate	0.35	0.263	0.34	0.243	0.38	0.198
Obesity rate	0.32	0.765	0.58	0.568	0.68	0.467
Unemployment rate	2.05	** 0.001	2.10	** 0.000	2.28	** 0.000
Uninsured rate	−0.25	0.689	−0.46	0.438	−0.67	0.260
NP abundance	0.12	0.160	0.13	0.111	0.13	0.106

NP: nurse practitioner; *: *p* < 0.05; **: *p* < 0.01.

**Table 3:** Coefficients of the explanatory variables in SLM.

	July 15		August 15		September 15	
	Coef.	*p* Value	Coef.	*p* Value	Coef.	*p* Value
Male percentage	10.70	* 0.015	9.28	** 0.008	9.65	** 0.002
White percentage	−0.07	0.911	−0.48	0.348	−0.02	0.966
Median age	0.04	0.962	0.41	0.537	0.03	0.960
College degree rate	0.44	0.372	0.22	0.583	0.45	0.228
Obesity rate	−3.16	0.076	−2.09	0.143	−0.25	0.846
Unemployment rate	2.83	** 0.003	1.93	* 0.011	2.19	** 0.004
Uninsured rate	1.75	0.081	0.64	0.419	0.12	0.875
NP abundance	0.07	0.640	0.03	0.801	0.07	0.490
	**October 15**		**November 15**		**December 15**	
	**Coeff.**	***p* Value**	**Coeff.**	***p* Value**	**Coeff.**	***p* Value**
Male percentage	12.81	** 0.000	13.30	** 0.000	13.16	** 0.000
White percentage	0.18	0.646	0.66	0.077	1.21	** 0.001
Median age	0.26	0.604	0.11	0.815	−0.10	0.840
College degree rate	0.15	0.608	0.14	0.611	0.19	0.522
Obesity rate	−0.86	0.431	−0.52	0.618	−0.16	0.882
Unemployment rate	2.01	** 0.001	2.09	** 0.000	2.31	** 0.000
Uninsured rate	0.09	0.882	−0.14	0.815	−0.37	0.528
NP abundance	0.11	0.213	0.12	0.149	0.13	0.124

NP: nurse practitioner; *: *p* < 0.05; **: *p* < 0.01.

**Table 4:** Coefficients of the explanatory variables in the CAR model.

	July 15		August 15		September 15	
	Coef.	*p* Value	Coef.	*p* Value	Coef.	*p* Value
Male percentage	9.59	* 0.012	8.10	** 0.009	9.97	** 0.001
White percentage	0.08	0.901	−0.10	0.849	0.01	0.988
Median age	−0.04	0.953	0.23	0.700	0.07	0.908
College degree rate	0.32	0.500	0.00	0.568	0.30	0.422
Obesity rate	−3.13	0.065	−1.61	0.242	−1.09	0.402
Unemployment rate	2.82	** 0.002	1.99	** 0.008	2.17	** 0.003
Uninsured rate	2.16	* 0.026	0.79	0.317	0.43	0.568
NP abundance	0.08	0.565	0.03	0.784	0.05	0.613
	**October 15**		**November 15**		**December 15**	
	**Coeff.**	***p* Value**	**Coeff.**	***p* Value**	**Coeff.**	***p* Value**
Male percentage	11.39	** 0.000	12.01	** 0.000	11.97	** 0.000
White percentage	0.33	0.445	0.71	0.079	1.18	** 0.003
Median age	0.12	0.813	0.00	0.996	−0.17	0.727
College degree rate	0.33	0.299	0.32	0.275	0.35	0.228
Obesity rate	0.24	0.821	0.53	0.598	0.66	0.512
Unemployment rate	2.02	** 0.001	2.07	** 0.000	2.27	** 0.000
Uninsured rate	−0.22	0.728	−0.44	0.461	−0.64	0.279
NP abundance	0.13	0.146	0.13	0.096	0.14	0.091

NP: nurse practitioner; *: *p* < 0.05; **: *p* < 0.01.

**Table 5:** Summary of the results from the GWR model fitting.

July 15th					
Covariates	Min.	Q1.	Median	Q3	Max.
Male percentage	6.73	7.68	8.11	8.50	8.98
White percentage	−0.59	−0.36	−0.20	−0.02	0.21
Median age	−0.67	−0.15	0.09	0.34	0.71
College degree rate	0.64	0.71	0.75	0.80	0.83
Obesity rate	−0.81	−0.49	−0.32	−0.10	0.22
Unemployment rate	1.82	2.15	2.36	2.66	3.18
Uninsured rate	0.48	0.76	0.93	1.20	1.58
NP abundance	0.17	0.20	0.21	0.23	0.24
**August 15th**					
Covariates	Min.	Q1.	Median	Q3	Max.
Male percentage	7.11	7.33	7.44	7.60	7.85
White percentage	−0.67	−0.65	−0.63	−0.61	−0.59
Median age	0.32	0.41	0.44	0.50	0.57
College degree rate	0.48	0.49	0.49	0.50	0.50
Obesity rate	−0.06	−0.05	−0.03	−0.01	0.02
Unemployment rate	1.57	1.64	1.69	1.75	1.85
Uninsured rate	−0.05	−0.01	0.01	0.05	0.10
NP abundance	0.12	0.12	0.12	0.12	0.13
**September 15th**					
Covariates	Min.	Q1.	Median	Q3	Max.
Male percentage	9.28	9.44	9.55	9.69	9.90
White percentage	−0.43	−0.38	−0.37	−0.35	−0.33
Median age	0.20	0.28	0.31	0.36	0.43
College degree rate	0.42	0.43	0.44	0.44	0.45
Obesity rate	−0.07	−0.05	−0.04	−0.02	0.01
Unemployment rate	1.77	1.83	1.87	1.93	2.02
Uninsured rate	−0.22	−0.17	−0.14	−0.11	−0.05
NP abundance	0.12	0.12	0.13	0.13	0.13
**October 15th**					
Covariates	Min.	Q1.	Median	Q3	Max.
Male percentage	11.37	11.58	11.69	11.82	12.05
White percentage	0.07	0.11	0.13	015	0.17
Median age	0.20	0.24	0.27	0.29	0.35
College degree rate	0.30	0.31	0.32	0.33	0.33
Obesity rate	0.29	0.31	0.33	0.36	0.39
Unemployment rate	1.81	1.86	1.89	1.94	2.00
Uninsured rate	−0.31	−0.27	−0.25	−0.22	−0.17
NP abundance	0.16	0.16	0.16	0.16	0.17
**November 15th**					
Covariates	Min.	Q1.	Median	Q3	Max.
Male percentage	11.96	12.15	12.24	12.37	12.57
White percentage	0.57	0.60	0.63	0.65	0.68
Median age	0.03	0.08	0.11	0.14	0.20
College degree rate	0.27	0.28	0.29	0.30	0.30
Obesity rate	0.53	0.55	0.57	0.59	0.64
Unemployment rate	1.91	1.95	1.98	2.03	2.09
Uninsured rate	−0.51	−0.47	−0.45	−0.43	−0.39
NP abundance	0.16	0.17	0.17	0.17	0.17
**December 15th**					
Covariates	Min.	Q1.	Median	Q3	Max.
Male percentage	12.03	12.19	12.29	12.43	12.62
White percentage	1.14	1.18	1.21	1.23	1.27
Median age	−0.18	−0.13	−0.10	−0.07	−0.02
College degree rate	0.28	0.29	0.30	0.31	0.32
Obesity rate	0.68	0.70	0.72	0.74	0.79
Unemployment rate	2.16	2.20	2.23	2.27	2.33
Uninsured rate	−0.69	−0.65	−0.63	−0.60	−0.57
NP abundance	0.16	0.17	0.17	0.17	0.17

NP: nurse practitioner.

**Table 6:** Summary of models’ AIC values over time.

	SEM	SLM	CAR	GWR
July 15	61.59	58.72	59.39	51.24
August 15	41.47	36.64	39.81	34.49
September 15	35.62	30.67	35.20	25.64
October 15	17.00	11.96	17.42	5.31
November 15	11.52	6.68	11.94	−0.54
December 15	10.74	8.31	11.13	−1.48

## Data Availability

The data presented in this study are available on request from the corresponding author.
